# Risk factors for wasting among hospitalised children in Nepal

**DOI:** 10.1186/s41182-022-00461-0

**Published:** 2022-09-16

**Authors:** Aiko Inoue, Bhim Gopal Dhoubhadel, Dhruba Shrestha, Ganendra Bhakta Raya, Yumiko Hayashi, Sudeep Shrestha, Tansy Edwards, Christopher Martin Parry, Koya Ariyoshi, Sharon Elizabeth Cox

**Affiliations:** 1 Department of Global Health, School of Tropical Medicine and Global Health (TMGH), Nagasaki University, 1-12-4 Sakamoto, Nagasaki, 852-8523 Japan; 2 Department of Respiratory Infections, Institute of Tropical Medicine, Nagasaki University, Nagasaki, Japan; 3 Siddhi Memorial Hospital, Bhaktapur, Nepal; 4 Clinical Sciences, Liverpool School of Tropical Medicine, Liverpool, UK; 5 London School of Hygiene & Tropical Medicine, London, UK; 6 Department of Clinical Medicine, Institute of Tropical Medicine, Nagasaki University, Nagasaki, Japan

**Keywords:** Malnutrition, Wasting, Children, Nepal, Earthquake, Hospital

## Abstract

**Background:**

Malnutrition has various adverse effects in children. This study aimed to determine risk factors for malnutrition among hospitalised children, changes in nutritional status at admission and discharge and effects of use of systematic anthropometric measurement in identification of malnutrition.

**Methods:**

We enrolled 426 children, aged between 6 months and 15 years, admitted to Siddhi Memorial Hospital, Bhaktapur, Nepal, from November 2016 to June 2017. Anthropometric measurements were performed at the time of admission and discharge. Risk factors were assessed by multivariable logistic regression models.

**Results:**

Median age of children was 26 months (IQR: 13–49), and males were 58.7%. The prevalence of wasting was 9.2% (39/426) at admission and 8.5% (36/426) at discharge. Risk factors associated with wasting at admission were ethnic minority (aOR: 3.6, 95% CI 1.2–10.8), diarrhoeal diseases (aOR = 4.0; 95% CI 1.3–11.8), respiratory diseases (aOR: 3.4, 95% CI 1.4–8.1) and earthquake damage to house (aOR = 2.6; 95% CI 1.1–6.3). Clinical observation by care providers identified only 2 out of 112 malnutrition cases at admission and 4 out of 119 cases at discharge that were detected by the systematic anthropometric measurement.

**Conclusions:**

Ethnic minority, diarrhoeal diseases, respiratory infections and house damage due to the earthquake were risk factors associated with wasting. Systematic anthropometric examination can identify significantly more malnourished children than simple observation of care providers.

## Introduction

Undernourished children suffer not only from insufficient physical and mental growth, but also have a high susceptibility to the infectious diseases and have a high risk of death. The UNICEF–WHO–World Bank Group Joint Malnutrition Estimates shows that the 45.4 million under 5 children suffered from wasting in 2020 [1]. Primary risk factors for undernutrition are poverty, lack of adequate food, recurrent illnesses, inappropriate feeding practices and lack of care and poor hygiene [2].

Although hospitalised children are at high risk of malnutrition, they are not routinely assessed for wasting at the time of admission [3]. Knowing nutritional status can help clinical management of the children as poor nutritional status is associated with an increased length of hospital stay and a worse prognosis for both communicable and non-communicable diseases [4, 5]. Wasting in hospitalised patients is a neglected issue, and children who are discharged with poor nutritional status are more likely to be re-admitted and have an increased risk of post-discharge mortality.[6].

In 2015 Nepal suffered two major earthquakes that killed about 8900 people along with damaging thousands of houses, health centres and food storage buildings. Natural disasters, including earthquakes, can put children at risk for undernutrition [7, 8]. Bhaktapur was one of the 14 districts, which was affected heavily by the earthquakes. We conducted this nutritional survey in Siddhi Memorial Hospital (SMH), Bhaktapur after 1 year of the earthquake to assess the prevalence of undernutrition among paediatric patients, which was unknown before. It was important as we could detect undernourished children and provide them appropriate treatment and counselling while they were in the hospital. We carried out the survey by introducing systematic anthropometric measurements to the hospital. In addition, we quantified the disparity between systematic anthropometric measurements and the care providers’ detection and assessed risk factors associated with wasting at the time of admission.

## Methods

### Study design

This study was a cross-sectional study (serial surveys) in paediatric patients newly admitted to the paediatric ward in Siddhi Memorial Hospital (SMH), Bhaktapur, Nepal. Cross-sectional data were collected at a point of their admission and discharge.

### Study setting

SMH is a 50-bed paediatric hospital in Bhaktapur district. It is the only paediatric hospital in the district where about 75,000 children (age < 15 years) live (https://dccbhaktapur.gov.np/en/brief-introduction/). SMH provides medical services in the following facilities: paediatric out-patient department, paediatric in-patient department, intensive care unit, emergency department, gynecology and obstetrics department and dental department. There are approximately 15,000 visits to the out-patient department and 1500 admissions to the in-patient department each year. The in-patient department has 50 beds. This hospital provides specialised care for relatively severe paediatric patients as it runs in-patient department and intensive care units, and patients out of Bhaktapur district also come for in-patient services.

### Subjects and recruitment method

All patients who were admitted to the paediatric in-patient department in SMH from 17th November 2016 to 30th June 2017 were approached for the recruitment. Among them, those whose reported age ranged from 6 months to 191 months (15 years) were eligible, and their parents/guardian were counselled for the enrolment of their children in the study. We excluded those whose parents or guardians did not give a written consent to take part in the study. Children with a severe clinical condition, such as requiring respiratory support, immobilised, severe burns, fractures, abdominal trauma or congenital syndromes (e.g., Down syndrome, congenital cardiac diseases) were excluded from the enrolment. The estimated sample size was calculated as 340 to detect a 5% difference in the prevalence of wasting (*z*-score < − 2 of weight-for-length or weight-for-height, or BMI) compared to the national average of 11% (in children < 5 years), computed by using single-sample *z*-test of proportion with power of 80% and significance of 5%.

### Case definition of wasting

Moderate wasting was defined as a weight-for-height/length *z*-score (WHZ) between -3 and -2 SD, and severe wasting was defined as WHZ below − 3 SD (WHO, 2009) or MUAC less than 115 mm for children less than 60 months. For children aged over 60 months, moderate wasting was defined as BMI for age *z*-score between − 3 and − 2 SD, and severe wasting was defined as below − 3 SD of the same indicator [9].

### Enrolment and anthropometric measurement

Research assistants were trained to perform anthropometric measurements. Two research assistants checked the ward-admission register once daily (including weekends), and when they found newly hospitalised patients, their parents or guardians were asked if their children could take part in the study. After obtaining written consent from them (and written assent for those whose children are 10 years or more), they were interviewed using structured questionnaires, using the Nepali language. The questionnaire includes the information about demographic detail of the child (birthday, ethnic group, religion, place of living, etc.), socioeconomic status of the household, earthquake effects, immunisation history, usual feeding history, recent dietary intake, medical record, and lab test results. Home damage was defined as any damage due to earthquake to their residence reported by the parents. The results of the haematological test were obtained from laboratory results sheet and the clinical diagnosis was obtained from medical records of the patient. After interviewing the parents or guardians, participating children were measured for weight, height (or length for babies < 2 years) and MUAC by the trained research nurses. Seca 833 (https://www.seca.com/) was used for measuring weight scale for infant and Seca 803 for adults; the minimum display units of them were 10 g and 100 g, respectively. A stadiometer (Seca 417) was used for measuring length/height that had minimum display unit of 1 mm. Weights for diapers and/or clothes (40–450 g) were subtracted according to age group of children. In normal condition, their weight and height were measured in standing posture using a stadiometer. When children could not maintain a standing posture, weight was measured by holding the child by the parent or guardian and then subtracted the adult’s weight from the results. In case of dehydrated patients, weight was measured only after correction of dehydration. For height measurement in such cases, the results of arm span substituted, because arm span can serve as accurate and reliable surrogate measures of recumbent length and height [10]. Anthropometric measurements were conducted again on the day of hospital discharge. Laboratory results and diagnosis were updated if applicable. Clinical diagnosis of nutritional status was made by treating doctors by looking at the children and general physical examination during the ward round which occurred every day.

### Data analysis

The anthropometric measurement and questionnaire survey data were entered into Epi Info (CDC, Atlanta, USA) software. The dataset was imported to STATA Version 15.0 (StataCorp, TX, USA) for further analysis. The sociodemographic characteristics of participants were described using descriptive statistics. Ethnicities were classified according to their prevalence in the hospitalised children. The first five ethnicities with higher prevalence were reported separately, and the remaining all were grouped as “Others”. Proportions were compared by using Chi-squared test. Inter-rater agreement for the clinical assessment and the measured assessment result was determined using the Kappa statistic. Calculated Kappa values of ≤ 0.20 are considered to reflect poor agreement, > 0.20 and ≤ 0.40 fair agreement, > 0.40 and ≤ 0.60 moderate agreement, > 0.60 and ≤ 0.80 good agreement and > 0.80 very good agreement. A logistic regression model was used to assess the risk factors. A priori variables (sex, child’s age and ethnicity) and variables whose *p* value < 0.2 in univariable analysis were included in the multivariable logistic regression model. The model was checked by the goodness of fit test.

### Ethical consideration

Ethical approvals were obtained from Nepal Health Research Council, Kathmandu, Nepal (Reg. no. 265/2016) and School of Tropical Medicine and Global Health (TMGH), Nagasaki University, Japan (Approval Ref no. 4, 2016). The study did not involve any invasive procedures; anthropometric measurements were performed with extreme cautions not to give any discomfort to the participants. The result of anthropometric measurement and short nutritional counselling were provided to the parents/guardian of the children by the research assistants. When the malnourished children were found, their parents or guardians were introduced to the out-patient therapeutic nutrition centre or hospital nutrition facility according to their severity.

## Results

**Fig. 1:**
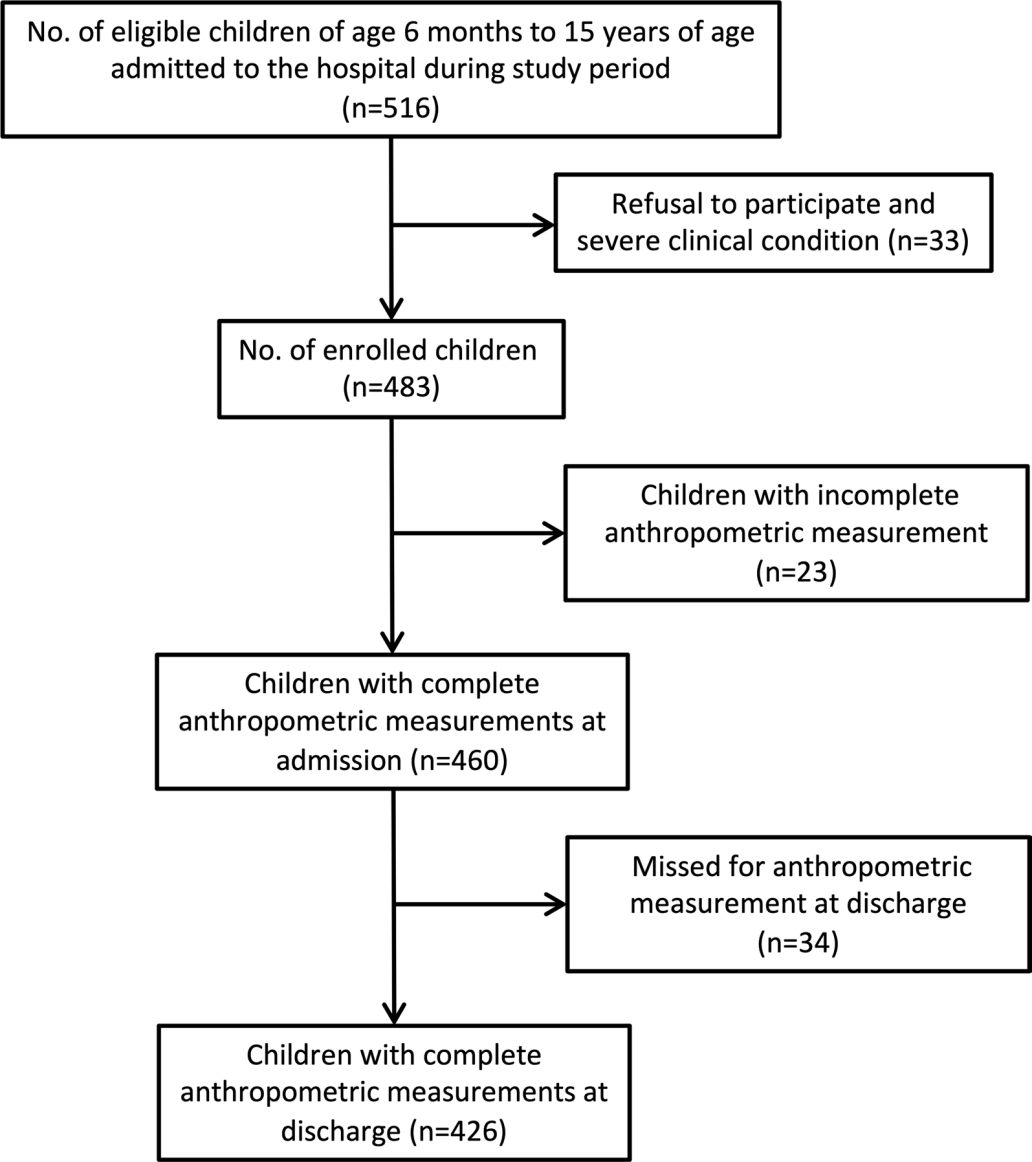
Flowchart showing the number of children from admission to enrolment

A total of 830 patients were admitted to the paediatric general ward in SMH during the study period. Among them, 516 children were eligible, and complete anthropometric measurement data could be collected from 426 children at admission and discharge (Fig. 1). The demographic characteristics of the children are shown in Table 1. Median age was 26 months (IQR: 13–49), and 59.4% were male.

**Table 1:** Characteristics of children enrolled in the study

Characteristics	No. of children (*N* = 426)
Age (months)	
6–23	197 (46.3)
24–59	150 (35.2)
≥ 60	79 (18.5)
Sex	
Male	253 (59.4)
Female	173 (40.6)
Residence	
Bhaktapur	139 (32.6)
Out of Bhaktapur	287 (67.4)
Ethnicity	
Newar	152 (35.7)
Tamang	102 (23.9)
Chhettri	78 (18.3)
Brahmin	39 (9.2)
Magar	21 (4.9)
Others^#^	34 (8.0)
Number of children in family	
One	217 (50.9)
Two	158 (37.1)
≥ Three	51 (12.0)
Mother's age (years)	
< 20	8 (1.9)
20–34	385 (90.4)
≥ 35	33 (7.7)
Mother's education (*n* = 424)	
Primary school	127 (30.0)
Secondary school	134 (31.6)
University	163 (38.4)
Source of drinking water	
Public	103 (24.2)
Private	323 (75.8)
Home damaged due to earthquake	
No	135 (31.7)
Yes	291 (68.3)
Main diagnosis for hospital admission	
Respiratory infections	183 (43.0)
Diarrhoeal diseases	62 (14.5)
Others	181 (42.5)

^#^Others includes ethnic minorities: Rai, Bishwokarma, Damai, Nepali, Majhi, Sewa, Thakuri, Limbu, Dalit, Tharu, Puri, Giri, Bangali, Gurung, Sanyashi

Prevalence of wasting, underweight and stunting at the time of admission and discharge are shown in Table 2. There was no significant change in any nutritional parameter during the hospital admission. Among the 426 children, care providers, including doctors identified 2 cases of malnutrition (wasting or underweight or stunting) compared to 112 cases by the systematic anthropometric measurement at admission (% of agreement = 74.2%; Cohen’s *k* = 0.02) and 4 cases compared to 119 cases by the anthropometric measurement (% of agreement = 72.5%; Cohen’s *k* = 0.02) at discharge. The Cohen’s kappa shows the slight agreement. The systematic anthropometric measurement identified far more numbers of wasting cases than the simple observation of doctors and nurses. Out of 39 children who were identified as wasting at admission, 12 (30.8%) improved to be normal at discharge; while 387 children who were identified as not-wasting (normal) at admission, 9 (2.3%) deteriorated to be wasting at discharge. All malnourished children were given nutritional counselling at the hospital, and severely malnourished cases were referred to a nutritional rehabilitation centre.

**Table 2:** Nutritional status of children at the time of admission and at discharge in Siddhi Memorial Hospital

Nutritional parameter*	At admission *n* = 426 (%)	At discharge *n* = 426 (%)
Wasting (weight/height)		
Moderate	28 (6.6)	24 (5.6)
Severe	11 (2.6)	12 (2.8)
Underweight (weight/age)		
Moderate	56 (13.2)	48 (11.3)
Severe	9 (2.1)	13 (3.1)
Stunting (height/age)		
Moderate	61 (14.3)	63 (14.8)
Severe	20 (4.7)	22 (5.2)
Anaemia (*n* = 393)^#^	8 (2.0)	NA

^*^Moderate wasting was defined by weight/height *z*-score < − 2 to < − 3 for children < 5 years and BMI for age *z*-score < − 2 to − < 3 for children ≥ 5 years of age. Severe wasting was defined by weight/height *z*-score ≥ -3 for children < 5 years OR MUAC < 115 mm, and BMI for age *z*-score ≥ − 3 for children ≥ 5 years of age

^#^Anaemia was defined by Hb below normal range. Normal range of Hb are: 9.0–14.0 g/dL (for 6 months to 5 years); 11.5–15.5 g/dL (for 6 to 12 years); 13.0–16.0 g/dL (12 to 15 years/ male); 12.0–16.0 g/dL (12 to 15 years/female)

Univariable and multivariable analyses showed children from minor ethnic group (Others, aOR: 3.6, 95% CI 1.2–10.8), children from earthquake affected home (aOR: 2.6, 95% CI 1.1–6.3), and with respiratory diseases (aOR: 3.4, 95% CI 1.4–8.1) diarrhoea diseases (aOR: 4.0, 95% CI 1.3–11.8) were at risk of wasting (Table 3). Childrens’ age group, sex, residence, mother’s age, mother’s education and source of drinking water were not associated.

**Table 3:** Risk factors associated with wasting in hospitalised children after 2015 earthquake in Bhaktapur, Nepal

Characteristics	Wasted detected (*N* = 39)	Wasted not detected (*N* = 387)	Odds ratio (OR) 95% CI	*P*-value	Adjusted OR^#^	*P*-value
Age, months						
6–23	19 (48.7)	178 (46.0)	1.1 (0.4-2.7)	0.84	0.7 (0.2-1.8)	0.41
24–59	13 (33.3)	137 (35.4)	1.0 (0.4–2.6)	0.96	0.8 (0.3–2.2)	0.65
≥ 60	7 (18.0)	72 (18.6)	1		1	
Sex						
Male	22 (56.4)	231 (59.7)	0.9 (0.4-1.7)	0.69	0.9 (0.4-1.8)	0.70
Female	17 (43.6)	156 (40.3)	1		1	
Residence						
Bhaktapur	9 (23.1)	130 (33.6)	1			
Out of Bhaktapur	30 (76.9)	257 (66.4)	1.7 (0.8–3.7)	0.50		
Ethnicity						
Newar	11 (28.2)	141 (36.4)	1		1	
Tamang	6 (15.4)	96 (24.8)	0.8 (0.3–2.2)	0.67	0.6 (0.2–1.8)	0.42
Chhettri	10 (25.6)	68 (17.6)	1.9 (0.8–4.7)	0.17	1.6 (0.6–4.0)	0.33
Brahman	4 (10.3)	35 (9.0)	1.5 (0.4–4.9)	0.53	1.2 (0.4–4.2)	0.74
Magar	1 (2.6)	20 (5.2)	0.6 (0.1–5.2)	0.68	0.6 (0.1–5.2)	0.66
Others^%^	7 (18.0)	27 (7.0)	3.3 (1.2–9.3)	0.02	3.6 (1.2–10.8)	0.02
Mother's age (years)						
< 20	1 (2.6)	7 (1.8)	1.3 (0.2–11.2)	0.79		
20–34	37 (94.8)	348 (89.9)	1			
≥ 35	1 (2.6)	32 (8.3)	0.2 (0.03–2.2)	0.24		
Mother's education (*n* = 424)						
Primary school	12 (30.8)	115 (29.9)	1.4 **(**0.6–3.4)	0.40		
Secondary school	16 (41.0)	118 (30.7)	1.9 (0.8–4.2)	0.12		
University	11 (28.2)	152 (39.5)	1			
Source of drinking water						
Public	10 (25.6)	93 (24.0)	1			
Private	29 (74.4)	294 (76.0)	0.9 (0.4–2.0)	0.82		
Home damaged due to earthquake						
No	7 (18.0)	128 (33.1)	1		1	
Yes	32 (82.0)	259 (66.9)	2.3 (1.0–5.3)	0.06	2.6 (1.1–6.3)	0.03
Main diagnosis for hospital admission						
Respiratory infections	23 (59.0)	160 (41.3)	3.1 (1.4–7.1)	0.01	3.4 (1.4–8.1)	0.01
Diarrhoeal diseases	8 (20.5)	54 (14.0)	3.2 (1.1–8.9)	0.03	4.0 (1.3–11.8)	0.01
Others	8 (20.5)	173 (44.7)	1		1	

^#^Odds ratios were adjusted by the multivariable analysis; it was performed as keeping sex and age as a priori variables and other variables whose p-value were < 0.2 in the univariable analysis

^%^Others includes ethnic minorities: Rai, Bishwokarma, Damai, Nepali, Majhi, Sewa, Thakuri, Limbu, Dalit, Tharu, Puri, Giri, Bangali, Gurung, Sanyashi

There were 34 children from the ethnic minorities, which included Rai, Bishwokarma, Damai, Nepali, Majhi, Sewa, Thakuri, Limbu, Dalit, Tharu, Puri, Giri, Bangali, Gurung, Sanyashi. Newars were the major ethnic group in the community, which constituted 35.7% of children in the study. Comparisons between the minor ethnic group with the Newars are shown in Table 4. Children in the minority group were younger (78.4% vs 42.1% in 6–23 months age group), a higher proportion of them were from out of Bhaktapur, a lower proportion of their mothers went to university, had less private water source, and had higher proportions of wasting and underweight than those of children from the Newars (Table 4). This shows there were significant differences in socioeconomic and nutritional characteristics of children who came from the minor and the major ethnic groups.

**Table 4:** Comparing characteristics of children from a major ethnic group (Newars) and a group of the ethnic minorities

Characteristics	Newars (*N* = 152)	Ethnic minorities (*N* = 34)	*P*-value
Age (months)			
6–23	64 (42.1)	27 (78.4)	0.01
24–59	57 (37.5)	6 (17.7)	
≥ 60	31 (20.4)	1 (2.9)	
Sex			
Male	98 (64.5)	20 (58.8)	0.54
Female	54 (35.5)	14 (41.2)	
Residence			
Bhaktapur	74 (48.7)	8 (23.5)	0.01
Out of Bhaktapur	78 (51.3)	26 (76.5)	
Number of children in family			
One	85 (55.9)	19 (55.9)	0.61
Two	50 (32.9)	13 (38.2)	
≥ Three	17 (11.2)	2 (5.9)	
Mother's age (years)			
< 20	1 (0.7)	1 (2.9)	0.45
20–34	138 (90.8)	31 (91.2)	
≥ 35	13 (8.5)	2 (5.9)	
Mother's education			
Primary school	32 (21.0)	13 (38.2)	0.01
Secondary school	41 (27.0)	13 (38.2)	
University	79 (52.0)	8 (23.6)	
Source of drinking water			
Public	37 (24.3)	15 (44.1)	0.02
Private	115 (75.7)	19 (55.9)	
Home damaged due to earthquake			
No	47 (30.9)	13 (38.2)	0.41
Yes	105 (69.1)	21 (61.8)	
Main diagnosis for hospital admission			
Respiratory infections	58 (38.2)	16 (47.1)	0.13
Diarrhoeal diseases	14 (9.2)	6 (17.6)	
Others	80 (52.6)	12 (35.3)	
Wasting	11 (7.2)	7 (20.6)	0.02
Underweight	16 (10.5)	10 (29.4)	0.01
Stunting	25 (16.5)	10 (29.4)	0.08

## Discussion

In this cross-sectional study, conducted 18 months after the 2015 Nepal Earthquake, the prevalence of wasting among hospital admitted children was 9.2% at admission and 8.5% at discharge. Children from minor ethnic groups, children with respiratory infections, diarrhoeal disease, and whose house was damaged due to the earthquake were associated with wasting. Care providers, including medical doctors in routine care could only identify 2 out of 112 children with malnutrition at admission and 4 out of 119 children with malnutrition at discharge. This corresponds to 98.2% of cases missed at admission and 96.6% of cases not identified at discharge.

The prevalence of malnutrition among the study population at admission was wasting 9.2%, stunting 19.0% and underweight 15.3% which were lower than the national average of wasting 10%, stunting 36% and underweight 27% [11]. This is because our study population is from the Kathmandu valley where probably the access to food is better than the rural areas of Nepal. The prevalence of wasting in hospital-based study conducted in Brazil, Romania and Vietnam report 16.1%, 17.7% and 19.0%, respectively [4, 12, 13]. Compared with the national average of prevalence of wasting 1.6% in Brazil, 3.3% in Romania and 5.7% in Vietnam, hospitalised children were more likely to have wasting than non-hospitalised children [14]. Studies from Tanzania and Burkina Faso report the prevalence of malnutrition in hospitals were comparable to the national averages [15, 16]. These findings are comparable with the findings obtained from our study.

Our risk factor analysis for wasting showed that the children from minority ethnic group, whose house was damaged due to the earthquake, and who had respiratory or diarrhoeal diseases were independently associated with wasting. The minority ethnic group consisted of various minorities, such as Rai, Bishwokarma, Damai, Nepali, Majhi, Sewa, Thakuri, Limbu, Dalit, Tharu, Puri, Giri, Bangali, Gurung, Sanyashi. When minority ethnic group was compared with the Newars group, a major ethnic group in Bhaktapur, we found a less proportion of the mothers in minority ethnic group had university education as compared to Newars group. The proportions of children with wasting and underweight were significantly higher in minority ethnic group than that of Newars group. Previous studies from Nepal have also shown that lower mother’s education and ethnic minority are associated with underweight and stunting [17, 18]. Similarly, studies in other countries have shown that lower mother’s education and minor ethnicity can be a risk of malnutrition [19, 20]. Therefore, it is important that public health programmes should consider a targeted approach for the children with mothers with lower educational attainment and as well as ethnic minorities in the community.

In this study, diarrhoeal diseases and respiratory infections were associated with malnutrition. Malabsorption and nutrient loss occur during diarrhoea, which results in the deterioration of nutritional status of children. Recurrent infections, such as intestinal parasitic infections and diarrhoeal diseases, are causes of undernutrition in children. There is a risk of a vicious cycle of diarrhoea and malnutrition: repeated attacks of diarrhoea and infections leads to weight loss and compromise a child’s nutritional status, which makes the child vulnerable to infections and cause further weight loss, eventually leading to severe malnutrition unless the cycle is stopped [21, 22]. Association of malnutrition and respiratory infections in children is well known, and the association increases the risk of death [23, 24]. Malnourished children have deranged immune system; therefore, viral and bacterial pathogens can easily cause various infections, including the respiratory infections. Therefore, it is important to suspect and treat respiratory infections in malnourished children [25].

Malnutrition is viewed as one of the five major adverse health impacts of climate change and disaster [26]. Several studies showed deterioration of nutritional status of children after a huge disaster, irrespective of disaster type. In this study, children whose houses had suffered damage were at higher risk of wasting compared to the children whose house got no damage. A nutritional survey conducted 1 year after the Wenchuan Earthquake, China, reports higher prevalence of malnutrition [27]. There can be various reasons behind the causes of malnutrition in natural disasters. Some of them include nutrition insecurity, loss of income, damage to crops and death of livestock [28, 29]. Pörtner et al. points out a prolonged stay in temporary shelters can be a risk for malnutrition as it is not a secure place for breastfeeding because of lack of privacy [8].

In this study, care providers, including medical doctors, could identify 2 cases of malnutrition out of 112 at the time of admission and 4 cases out of 119 cases at the time of discharge by clinical observation alone. Thus, just depending on the clinical observations, many cases of malnutrition can be missed despite the chance to be examined by health care providers; similar findings were observed in other studies too [30, 31]. The research team presented these findings to the doctors and administrative staff in the hospital. Later, the hospital started the systematic anthropometric measurement of all admitted children to screen for malnutrition.

This study has limitations. We could not enrol all the children who were admitted to hospital. Some children, who had serious conditions, including sepsis and severe pneumonia that might have partly contributed to malnutrition. The study could not be conducted over a period of a year; so, we could not analyse any variation of nutritional status according to seasons of admission. Similarly, we could not analyse micronutrient deficiencies.

In conclusion, this study found that malnutrition among hospitalised children was associated with ethnic minority, diarrhoeal diseases, respiratory infections, and earthquake damage to house. Malnutrition remains an unrecognised problem among hospital admitted children. Due to lack of the systematic anthropometric measurement at the hospital at the time of this study, health care providers did not identify most malnutrition cases. This was a missed opportunity for identification, treatment and prevention of malnutrition, which could have multifaceted effects on children’s physical, mental and social developments. Therefore, health care facilities should provide the systematic anthropometric measurement to all hospital admitted children.

## Data Availability

De-identified data of this study are available upon reasonable request from the corresponding author.

## Abbreviations

BMI: Body mass index
CDC: Centers for Disease Control and Prevention, USA
MUAC: Mid upper-arm circumference
SMH: Siddhi Memorial Hospital
UNICEF: The United Nations Children's Fund
WHO: World Health Organization
WHZ: Weight-for-height Z-score
